# Cutaneous *Staphylococcus aureus* Infections in Renal Edema Across Kidney Disease and the Intensive Care Unit: Pathophysiological Mechanisms, Clinical Implications, and Therapeutic Challenges

**DOI:** 10.3390/ijms27136038

**Published:** 2026-07-05

**Authors:** Mariana-Emilia Caragea, Daniel Cosmin Caragea, Marius Bogdan Novac, Lidia Boldeanu, Mohamed-Zakaria Assani, Dragoș Forțofoiu, Vlad Pădureanu, Mihail Virgil Boldeanu, Dragoș-Marian Popescu, Cristin Constantin Vere

**Affiliations:** 1 Doctoral School, University of Medicine and Pharmacy of Craiova, 200349 Craiova, Romania; mariana.emilia77@yahoo.com (M.-E.C.); mohamed.assani@umfcv.ro (M.-Z.A.); dragos.fortofoiu@umfcv.ro (D.F.); 2 Department of Nephrology, Faculty of Medicine, University of Medicine and Pharmacy of Craiova, 200349 Craiova, Romania; daniel.caragea@umfcv.ro; 3 Department of Anesthesiology and Intensive Care, University of Medicine and Pharmacy of Craiova, 200349 Craiova, Romania; 4 Department of Microbiology, Faculty of Medicine, University of Medicine and Pharmacy of Craiova, 200349 Craiova, Romania; 5 Department of Immunology, Faculty of Medicine, University of Medicine and Pharmacy of Craiova, 200349 Craiova, Romania; mihail.boldeanu@umfcv.ro; 6 Department of Internal Medicine, Faculty of Medicine, University of Medicine and Pharmacy of Craiova, 200349 Craiova, Romania; vlad.padureanu@umfcv.ro; 7 Department of Extreme Conditions Medicine, Faculty of Medicine, University of Medicine and Pharmacy of Craiova, 200349 Craiova, Romania; dragos.popescu@umfcv.ro; 8 Department of Gastroenterology, University of Medicine and Pharmacy of Craiova, 200349 Craiova, Romania; vere_cristin@yahoo.com

**Keywords:** *Staphylococcus aureus*, MRSA, skin and soft tissue infections, renal edema, nephrotic syndrome, chronic kidney disease, cutaneous infection, virulence factors, biofilm

## Abstract

*Staphylococcus aureus*, particularly methicillin-resistant *S. aureus* (MRSA), remains a leading cause of skin and soft tissue infections (SSTIs) worldwide. Patients with renal edema, including those with nephrotic syndrome and chronic kidney disease (CKD), and critical illness, are particularly susceptible because of barrier dysfunction, immune impairment, and altered antimicrobial pharmacokinetics. This narrative review examines the mechanisms linking renal edema to increased susceptibility to cutaneous *S. aureus* infection and discusses their diagnostic and therapeutic implications. Three interconnected pathophysiological pathways appear central to this susceptibility: disruption of the cutaneous barrier, nephrotic and uremic immune dysfunction, and impaired lymphatic immune surveillance. These abnormalities facilitate bacterial colonization, and invasion, while *S. aureus* further exploits the renal host through adhesins, toxins, biofilm formation, and immune-evasion mechanisms. The review also highlights the challenges of managing severe staphylococcal infections in patients with kidney disease and critical illness, where augmented renal clearance, expanded volume of distribution, extracorporeal renal support, and fluctuating renal function may substantially influence antimicrobial exposure. Current management requires early recognition, source control, individualized antimicrobial selection, renal-adapted dosing, therapeutic drug monitoring, and antimicrobial stewardship. Although emerging anti-virulence and immunomodulatory strategies show promise, most remain at the preclinical or early translational stage. Overall, renal edema should be regarded as a biologically active modifier of host–pathogen interactions that contributes to increased susceptibility to cutaneous *S. aureus* infection across the spectrum of kidney disease.

## 1. Introduction

*Staphylococcus aureus* is one of the most clinically significant bacterial pathogens known to medicine, responsible for a broad spectrum of disease, from superficial skin pustules to fatal septicemia. Its dual capacity to colonize healthy individuals asymptomatically and to cause invasive, life-threatening illness in vulnerable hosts has sustained its prominence in infectious disease over multiple decades. Global meta-analysis data published in 2024 report a worldwide prevalence of *S. aureus* colonization of approximately 24.8% (95% CI, 20.4–29.3%), with methicillin-resistant *S. aureus* (MRSA) accounting for approximately 5.8% of all isolates globally [1].

*S. aureus* is the predominant pathogen in skin and soft tissue infections (SSTIs) in the United States, with clinical presentations ranging from superficial impetigo and folliculitis to monomicrobial necrotizing fasciitis. In 2023, SSTIs attributable to *S. aureus* were associated with an age-standardized all-cause mortality rate of 0.5 per 100,000 globally [2]. Moreover, in 2025, *S. aureus* was associated with more than 1 million deaths worldwide (range 816,000–1,470,000), cementing its position as the leading cause of bacterial infection-related mortality [3].

Although renal edema is classically associated with nephrotic syndrome, it is also frequently encountered in advanced chronic kidney disease, dialysis-dependent kidney disease, cardiorenal syndromes, and other conditions characterized by sodium and water retention. Importantly, edema often coexists with systemic immune dysfunction, chronic inflammation, vascular abnormalities, and altered antimicrobial pharmacokinetics, creating a clinical environment that may favor bacterial colonization and infection. Consequently, the pathophysiological mechanisms discussed in this review may have broader relevance across multiple renal disorders beyond nephrotic syndrome alone [4,5,6,7,8,9].

Patients with CKD or acute glomerular pathology represent a group of particular vulnerability. CKD is characterized by progressive impairment of renal filtration, culminating in the accumulation of uremic toxins and a state of composite immune dysfunction. *S. aureus* exploits this diminished host defense on multiple levels—impaired neutrophil chemotaxis, defective phagocytosis, disrupted T-cell signaling, and reduced immunoglobulin production all converge to create a permissive environment for bacterial invasion and propagation [7].

The cutaneous barrier is further compromised by the mechanical and biochemical consequences of renal edema, a defining feature of nephrotic syndrome and advanced CKD. Skin tension, blistering, and exudate create portals of entry for *S. aureus*, while impaired lymphatic drainage disrupts the local immunological surveillance that would ordinarily contain superficial colonization [10,11].

Rather than a simple manifestation of fluid overload, renal edema creates a complex pathological microenvironment characterized by compromised structural barriers, dysregulated innate and adaptive immunity, impaired lymphatic clearance, and altered antimicrobial pharmacokinetics. These interconnected abnormalities may collectively facilitate the transition of *S. aureus* from asymptomatic colonizer to invasive pathogen.

This review synthesises the current evidence on these interlocking pathophysiological mechanisms, delineates the clinical spectrum of cutaneous staphylococcal infections in the renal patient, appraises therapeutic strategies adapted to the unique constraints of renal function impairment, and addresses the specific challenges of managing *S. aureus* infections in the intensive care unit (ICU)—where critical illness, dialysis-dependent pharmacokinetics, and staphylococcal virulence converge to create the most demanding management scenario in this patient population.

## 2. Literature Search Strategy

This article is a narrative review that synthesizes current evidence on the pathophysiological links among renal edema, kidney disease, and susceptibility to cutaneous *S. aureus* infection, as well as the associated diagnostic and therapeutic challenges.

A structured literature search was conducted using PubMed/MEDLINE, Scopus, and Google Scholar databases to identify relevant publications from January 2016 to December 2025. Additional landmark studies published before this period were included when considered essential for understanding key pathophysiological mechanisms or major advances in anti-staphylococcal therapy. Search terms included combinations of the following keywords: “*Staphylococcus aureus*”, “MRSA”, “skin and soft tissue infection”, “renal edema”, “chronic kidney disease”, “nephrotic syndrome”, “dialysis”, “immune dysfunction”, “lymphatic dysfunction”, “critical illness”, “intensive care unit”, “augmented renal clearance”, “vancomycin pharmacokinetics”, and “anti-virulence therapy”.

Priority was given to clinical guidelines, systematic reviews, meta-analyses, prospective and retrospective clinical studies, translational investigations, and landmark experimental studies addressing host–pathogen interactions, renal-associated immune dysfunction, antimicrobial pharmacokinetics, and infection-related outcomes. Reference lists of selected articles were also manually screened to identify additional relevant publications.

Studies not published in English, conference abstracts without full-text availability, duplicate reports, and publications lacking direct relevance to cutaneous *S. aureus* infection, renal disease, or edema-associated pathophysiology were excluded. The selected evidence was synthesized narratively, with particular emphasis on mechanistic pathways, clinical implications, therapeutic considerations, and emerging treatment strategies relevant to patients with kidney disease and critical illness.

## 3. Renal Edema as a Gateway to Cutaneous Infection: From Hemodynamics to Immune Failure

For clarity, the term “renal edema” is used throughout this review as an umbrella term encompassing edema associated with nephrotic syndrome, CKD, dialysis-dependent kidney disease, and other renal conditions characterized by clinically significant fluid accumulation. More specific terms (e.g., nephrotic edema or CKD-associated edema) are used only when referring to particular pathophysiological contexts or study populations.

This section examines the mechanistic basis of enhanced cutaneous susceptibility to *S. aureus* in patients with renal disease. The following subsections address, in sequence: the haemodynamic and structural mechanisms underlying oedema formation in nephrotic syndrome and CKD (Section 3.1); the specific patterns of immune dysfunction—nephrotic immunodeficiency—that reduce host capacity to resist staphylococcal invasion (Section 3.2); the contribution of uremic toxin accumulation to innate and adaptive immune failure (Section 3.3); the role of lymphatic dysfunction in abolishing regional immunological surveillance (Section 3.4); and the broader spectrum of cutaneous manifestations through which CKD creates the integumentary conditions permissive for infection (Section 3.5).

### 3.1. Mechanisms of Edema Formation in Renal Disease

Edema is one of the cardinal features of nephrotic syndrome, occurring alongside hypoalbuminemia, proteinuria, and dyslipidemia. Its pathogenesis has historically been framed by two competing hypotheses. The classical “underfill” model postulates that urinary albumin loss reduces plasma oncotic pressure, causing fluid to shift into the interstitium. The resulting intravascular hypovolaemia triggers neurohormonal compensatory mechanisms, activation of the renin–angiotensin–aldosterone system, release of antidiuretic hormone, and sympathetic nervous system stimulation, which increase renal sodium and water retention, perpetuating edema [5]. Conversely, the “overfill” model, supported by direct measurements of circulating volume in many adult nephrotic patients, proposes that primary intrarenal sodium retention, independent of oncotic changes, is the driving mechanism, leading to plasma volume expansion and peripheral edema. Contemporary evidence suggests that both mechanisms operate, with their relative contributions varying by underlying etiology, glomerular pathology, and disease stage [5].

The glomerular filtration barrier normally restricts protein passage through the coordinated action of the glomerular endothelium, the glomerular basement membrane, and podocyte foot processes with their slit diaphragm architecture. Loss of podocyte function—through effacement of foot processes or disruption of the slit diaphragm—is the shared structural endpoint of most nephrotic conditions, including minimal change disease, focal segmental glomerulosclerosis, and membranous nephropathy [12,13].

A clinically important but underappreciated consequence of sustained edema is its direct effect on the integumentary system. Edema increases skin tension, mechanical blistering, skin breakage, and extrusion of proteinaceous exudate—all of which compromise the structural integrity of the cutaneous barrier and create a culture medium for bacterial colonization and infection [10]. In severe or longstanding edema, skin weeping and maceration further reduce local concentrations of antimicrobial peptides and disrupt the acid mantle, thereby compounding susceptibility to *S. aureus*.

**Figure 1:**
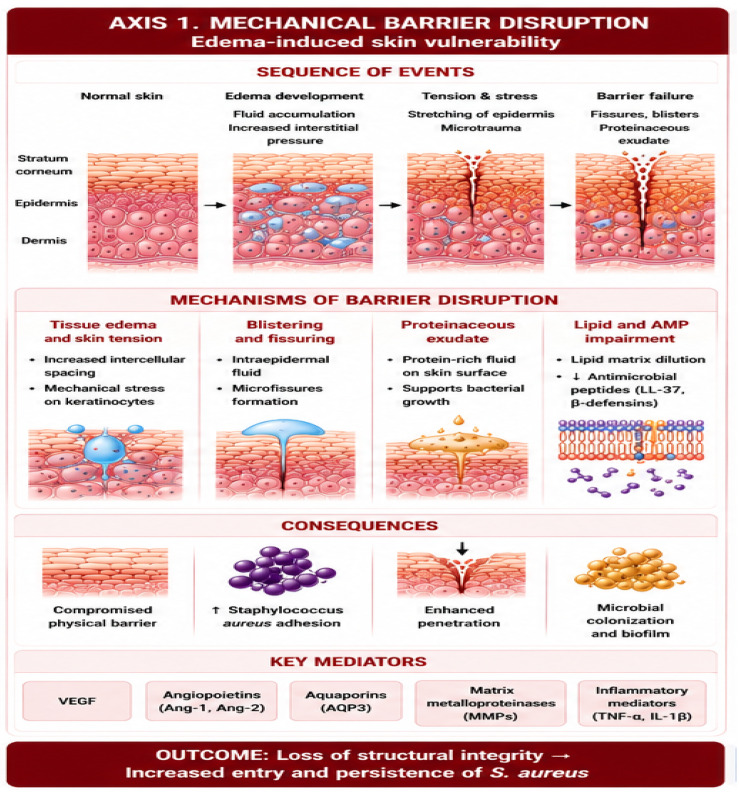
Edema-induced mechanical barrier disruption as a facilitator of cutaneous *S. aureus* infection (Figure created in Canva, https://www.canva.com; accessed on 9 June 2026). Mechanical barrier disruption. Progressive interstitial fluid accumulation increases skin tension and mechanical stress, promoting epidermal stretching, blistering, fissuring, and microtrauma. Protein-rich exudate on the skin surface promotes bacterial adherence and growth, while the dilution of lipid barrier components and antimicrobial peptides further compromises cutaneous defenses. These changes facilitate bacterial penetration, persistent colonization, and biofilm formation. Solid black arrows represent the direction of the mechanistic pathway linking sequential events. Upward arrows (↑) indicate increased expression, activation, or pathological enhancement, whereas downward arrows (↓) denote decreased function, depletion, or impairment.

Taken together, edema-induced epidermal stretching, fissuring, blister formation, proteinaceous exudation, and impairment of lipid and antimicrobial peptide defenses create a permissive microenvironment for bacterial colonization and invasion. The principal mechanisms through which renal edema compromises cutaneous barrier integrity and promotes susceptibility to *S. aureus* infection are summarized in Figure 1.

### 3.2. Nephrotic Immunodeficiency: Mechanisms and Consequences

Nephrotic syndrome is associated with a specific pattern of immune dysfunction—sometimes termed “nephrotic immunodeficiency”—that substantially increases susceptibility to encapsulated bacteria and, notably, to *S. aureus* [10]. The immune dysfunction associated with nephrotic syndrome is multifactorial and extends beyond simple urinary protein loss. Both innate and adaptive immune pathways are disrupted by interrelated mechanisms, including immunoglobulin depletion, complement loss, T-cell dysregulation, and treatment-related immunosuppression. The principal mechanisms contributing to nephrotic immunodeficiency, along with their clinical implications, are summarized in Table 1.

Among these mechanisms, urinary loss of immunoglobulins (Ig), particularly IgG, is perhaps the most clinically significant. IgG is required for opsonization of *S. aureus*, and its depletion reduces the efficiency of neutrophil-mediated bacterial killing. Concurrently, T-cell transformation dysfunction—characterized by an imbalance between Th2 and Th1 subsets and reduced regulatory T-cell (Treg) function—impairs the adaptive immune response to staphylococcal surface antigens, facilitating persistent colonization and recurrent infection [13].

### 3.3. Uremic Toxin-Mediated Immune Dysfunction in CKD

Beyond the specific mechanisms of nephrotic syndrome, the broader context of CKD imposes a distinct and compounding layer of immune dysfunction, driven primarily by the accumulation of protein-bound uremic retention solutes (PBURS)—including indoxyl sulfate, p-cresyl sulfate, and trimethylamine N-oxide. These molecules are derived from the gut microbiota’s metabolic activity and progressively accumulate as renal clearance diminishes [7].

PBURS impair neutrophil function at multiple levels. Uremic toxins inhibit neutrophil chemotaxis—the directed migration toward sites of infection—and reduce phagocytic capacity, such that neutrophils from CKD patients are significantly less effective at killing *S. aureus* than those from healthy controls. Critically, when neutrophils from CKD patients are cultured in a non-uremic medium, their phagocytic capacity is restored, confirming the direct causative role of circulating toxins rather than intrinsic neutrophil defects [7].

PBURS also impair the adaptive immune system: CKD patients exhibit premature T-cell aging (immunosenescence), defective dendritic cell function, and reduced cellular and humoral immune responses. This creates a state of paradoxical immune activation (chronic low-grade inflammation, endothelial damage) combined with immune incompetence (impaired antibacterial capacity), a combination uniquely permissive for *S. aureus* skin colonization and invasive infection [7,8].

**Figure 2:**
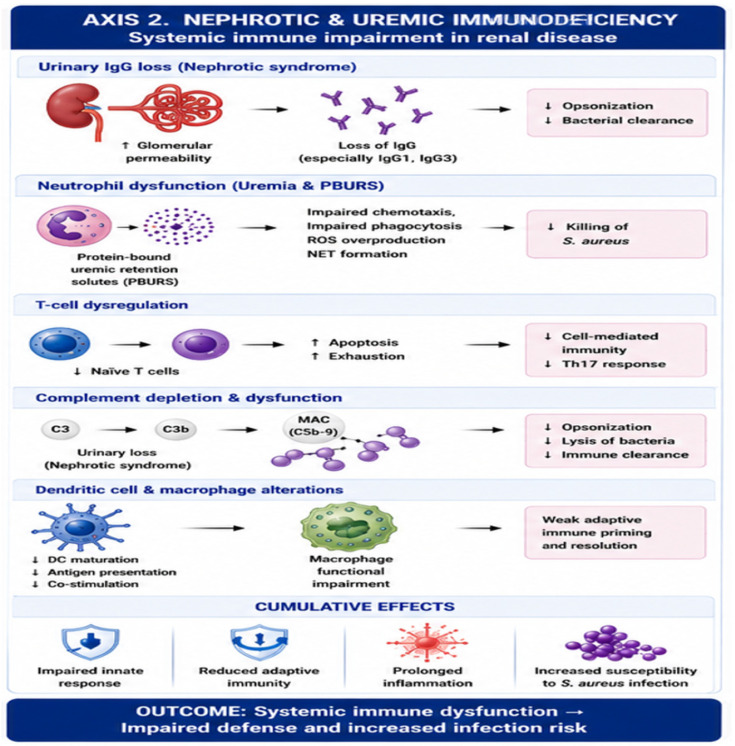
Nephrotic and uremic immunodeficiency as a driver of impaired anti-staphylococcal host defense (Figure created in Canva, https://www.canva.com; accessed on 9 June 2026). Nephrotic and uremic immunodeficiency. Urinary loss of immunoglobulins and complement components in nephrotic syndrome impairs opsonization and bacterial clearance. Concurrent accumulation of protein-bound uremic retention solutes (PBURS) contributes to neutrophil dysfunction, including impaired chemotaxis, phagocytosis, regulation of the oxidative burst, and neutrophil extracellular trap (NET) formation. T-cell dysregulation, dendritic cell dysfunction, and macrophage impairment collectively weaken adaptive immune priming and antibacterial host defense, resulting in increased susceptibility to *S. aureus* infection. Solid black arrows represent the direction of the mechanistic pathway linking sequential events. Upward arrows (↑) indicate increased expression, activation, or pathological enhancement, whereas downward arrows (↓) denote decreased function, depletion, impairment, or reduced antimicrobial defense.

The cumulative effect of urinary immunoglobulin loss, PBURS-mediated neutrophil dysfunction, complement abnormalities, T-cell dysregulation, and impaired antigen presentation is a profound reduction in anti-staphylococcal host defense. Rather than representing isolated immunological defects, these alterations collectively create a permissive environment for persistent colonization and invasive infection. The major pathways contributing to nephrotic and uremic immunodeficiency are illustrated in Figure 2.

### 3.4. Lymphatic Dysfunction and Regional Immunological Surveillance

The lymphatic system serves as the principal conduit for immune cell trafficking from peripheral tissues to regional lymph nodes, fulfilling a critical role in immunological surveillance. In renal edema, the lymphatic system is subjected to sustained overload because interstitial fluid volume exceeds its normal clearance capacity. This functional lymphatic insufficiency mirrors the pathophysiology of secondary lymphoedema and is associated with comparable immunological consequences [14]. In states of lymphatic dysfunction, defective lymphatic conduits prevent the homing of leukocytes and dendritic cells from peripheral tissues to draining lymph nodes. The resulting impairment of immune surveillance converts the edematous integument into an “immunologically vulnerable area”—a concept directly supported by the increased frequency of infections, including cellulitis and erysipelas, in edematous limbs [14]. Soft-tissue infections in this context carry a risk of progression to sepsis and may necessitate lifelong prophylactic antibiotic therapy in the most severe cases.

A 2023 experimental study by Cąkała-Jakimowicz et al. [11] formally demonstrated that interrupting lymphatic flow markedly worsens local cutaneous inflammation in the presence of commensal staphylococci. The combination of edema and bacterial inoculum induces severe local inflammation and delays the antibacterial protection processes in neighboring lymph nodes—establishing a mechanistic basis for the empirically observed susceptibility of edematous skin to staphylococcal superinfection [11].

**Figure 3:**
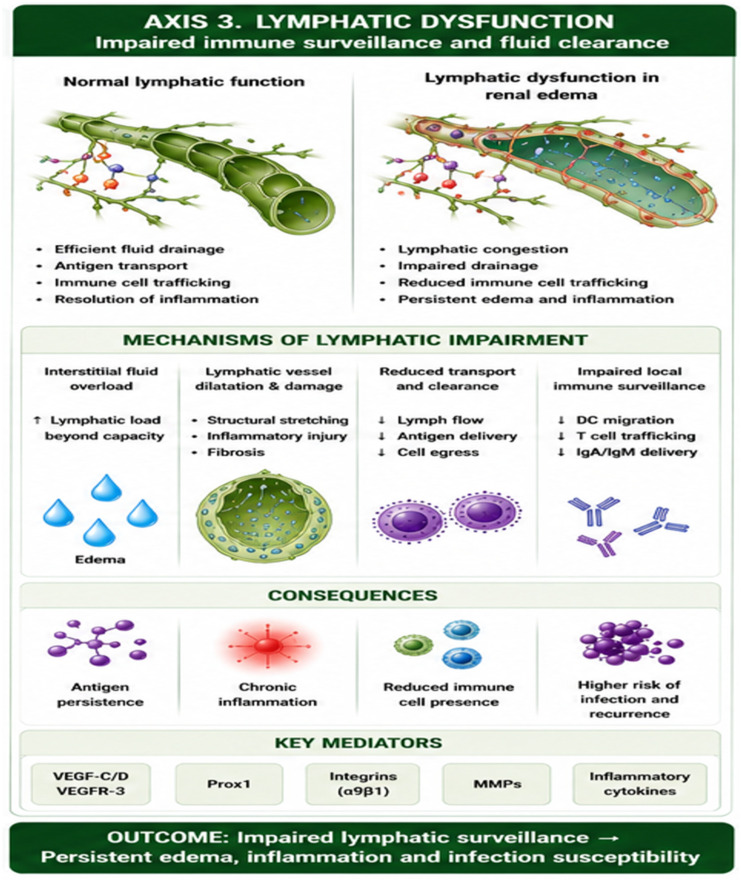
Lymphatic dysfunction and impaired immune surveillance in renal edema. Lymphatic dysfunction (Figure created in Canva, https://www.canva.com; accessed on 9 June 2026). Chronic interstitial overload exceeds lymphatic drainage capacity, leading to lymphatic congestion, structural vessel injury, and impaired immune cell trafficking. Reduced antigen transport and defective regional immune surveillance limit effective inflammatory resolution and facilitate persistent edema, chronic inflammation, recurrent infection, and bacterial persistence within edematous tissues. Together, these three interconnected mechanisms create a permissive microenvironment that facilitates *S. aureus* adhesion, immune evasion, tissue invasion, and recurrent cutaneous infection in patients with renal edema. Upward arrows (↑) indicate increased expression, activation, or pathological enhancement, whereas downward arrows (↓) denote decreased function, depletion, or impairment.

When lymphatic transport capacity is exceeded by sustained interstitial fluid accumulation, progressive lymphatic dysfunction develops, characterized by impaired drainage, reduced antigen transport, defective immune cell trafficking, and compromised local immune surveillance. The resulting persistence of edema and chronic inflammation creates a favorable microenvironment for bacterial survival and recurrent infection. The major pathways through which lymphatic impairment contributes to susceptibility to cutaneous *S. aureus* infection are summarized in Figure 3.

### 3.5. Cutaneous Manifestations in Chronic Kidney Disease: The Dermatological Interface

CKD produces a spectrum of cutaneous manifestations that reflect the systemic consequences of uremia, immune dysfunction, and altered dermal biology. A 2024 review by Arriaga Escamilla et al. [15] comprehensively cataloged these manifestations, identifying infectious complications as a major source of morbidity. Infections are predominantly caused by Gram-positive cocci—primarily *S. aureus* and coagulase-negative staphylococci (CONS)—with Gram-negative organisms, fungi, and polymicrobial infections occurring in immunocompromised subsets [15]. A defining feature of CKD-associated staphylococcal infections is the propensity of *S. aureus* and CONS to elaborate extracellular mucopolysaccharides, forming biofilms on compromised skin surfaces and vascular access sites. Biofilms confer marked resistance to both immune clearance and antibiotic penetration—contributing to the chronicity and recurrence that characterize infections in this population [15]. Key host-related risk factors for access-site and cutaneous infections in CKD patients include immunosuppression, prior bacteremia, poor hygiene, adjacent skin infection, high body mass index, iron overload, and, perhaps most importantly, hypoalbuminemia, which reduces both antimicrobial peptide transport and the nutritional substrate for wound healing [15].

The susceptibility of patients with renal edema to cutaneous *S. aureus* infection arises from the convergence of multiple interdependent pathophysiological processes rather than from a single isolated defect. Mechanical disruption of the edematous skin barrier facilitates bacterial entry; nephrotic and uremic immune dysfunction impairs both innate and adaptive antibacterial responses; and lymphatic insufficiency compromises regional immune surveillance and antigen clearance. Together, these mechanisms create a biologically permissive environment for persistent colonization, recurrent SSTIs, and invasive staphylococcal disease. The major mechanistic pathways linking renal edema to increased susceptibility to cutaneous *S. aureus* infection are summarized in Figure 1, Figure 2 and Figure 3.

Although these mechanisms are particularly evident in patients with clinically significant renal edema, many of the underlying abnormalities—including impaired innate immunity, complement dysfunction, lymphatic alterations, and pharmacokinetic variability—are also observed across broader CKD and dialysis populations. Therefore, renal edema may be viewed not only as a clinical manifestation of kidney disease but also as a marker of a more complex host environment predisposing to staphylococcal infection.

## 4. Virulence Mechanisms of *S. aureus* in the Cutaneous Context

The pathogenic potency of *S. aureus* in the skin derives from a precisely orchestrated, multi-layered virulence program. This section deconstructs that programme across three levels of analysis: the molecular mechanisms governing initial colonisation and host surface adhesion (Section 4.1); the panel of secreted toxins—alpha-toxin, Panton-Valentine Leukocidin, and exfoliative toxins—that directly damage cutaneous tissues and neutralise immune effectors (Section 4.2); and the transcriptional regulatory cascade (ArlRS/MgrA) that coordinates immune evasion in real time during skin infection and constitutes a compelling therapeutic target (Section 4.3). Throughout, the specific relevance of each mechanism to renal edema is highlighted.

### 4.1. Colonization Dynamics and Surface Adhesins

The pathogenic success of *S. aureus* in the skin begins with colonization—the establishment of a stable bacterial reservoir on the integumentary surface or within follicular structures, from which invasive infection can subsequently arise. Approximately 30% of the general population carries *S. aureus* asymptomatically in the anterior nares, with a transient proportion carrying *S. aureus* on the skin. Nasal, cutaneous, and oropharyngeal colonization serve as the primary reservoirs for both self-infection and transmission to susceptible contacts [16].

Surface adhesins mediate the initial attachment of *S. aureus* to host epithelial cells and extracellular matrix proteins. The clumping factors (ClfA and ClfB), fibronectin-binding proteins (FnBPA, FnBPB), and surface proteins such as SasG and IsdA facilitate adherence to corneocytes, fibronectin, fibrinogen, and loricrin—structural components of the stratum corneum. ClfB, in particular, has been identified as a key virulence factor in skin infection models and is under investigation as a vaccine target [16,17].

### 4.2. Secreted Toxins: Alpha-Toxin, PVL, and Exfoliative Toxins

*S. aureus* secretes a wide range of toxins that contribute to tissue injury, immune modulation, and bacterial dissemination. In patients with renal edema and kidney disease, the pathogenic consequences of these toxins may be amplified by concomitant barrier dysfunction and impaired host immune responses. The virulence toolkit operates synergistically; toxins with complementary mechanisms reinforce each other’s activity, creating functional redundancy that complicates therapeutic targeting [18].

The pathogenic impact of these secreted toxins may be particularly pronounced in patients with renal edema and kidney disease. Edema-associated barrier disruption facilitates deeper penetration of toxins into affected tissues, while nephrotic and uremic immune dysfunction reduces the host’s capacity to neutralize toxin-mediated injury and to efficiently eliminate invading bacteria. Consequently, toxin-driven tissue damage may contribute disproportionately to SSTI progression in renal patients compared with immunocompetent hosts.

Alpha-toxin (Hla) is a pore-forming cytotoxin that disrupts the integrity of epithelial and endothelial cell membranes, causing cell lysis and the release of pro-inflammatory danger signals. In a human skin explant model, alpha-toxin produced high tissue toxicity and complete loss of epithelial integrity, confirming its central role in the pathogenesis of superficial staphylococcal SSTIs [8]. Antibodies raised against alpha-toxin mitigated tissue damage in a concentration-dependent manner in the same model, supporting anti-toxin strategies as a potential adjunctive therapeutic approach [19].

Panton-Valentine Leukocidin (PVL) is a two-component pore-forming toxin (LukS-PV and LukF-PV) produced by fewer than 5% of *S. aureus* strains globally, but with a dramatically higher prevalence in community-associated MRSA (CA-MRSA) lineages. PVL forms cytolytic pores in the membranes of polymorphonuclear neutrophils (PMNs), monocytes, and macrophages, inducing apoptosis and lysing the primary cellular effectors of the early cutaneous innate immune response. PVL genes are detected in 93% of strains associated with furunculosis and in 55% of cellulitis isolates, compared with near-absent detection in hospital-acquired and device-related infections [20,21,22].

Exfoliative toxins (ETs)—including ETA, ETB, and ETD—are serine proteases that cleave desmoglein-1, the desmosomal cadherin responsible for intercellular adhesion in the superficial epidermis. Cleavage of desmoglein-1 produces the characteristic superficial skin cleavage of staphylococcal scalded skin syndrome (SSSS) and bullous impetigo. The clinical relevance of ETs extends to patients with renal disease: ET clearance depends on adequate renal function, and patients with CKD or AKI may accumulate ETs to locally or systemically toxic concentrations, even in the absence of bacteremia [18,23].

The pathogenic success of *S. aureus* in edematous and immunocompromised tissues depends on a coordinated repertoire of adhesins, toxins, immune evasion systems, and regulatory pathways. Several of these virulence determinants are of particular clinical relevance in patients with CKD and renal edema, due to impaired immune clearance and altered toxin handling. The major virulence factors implicated in cutaneous infection and their relevance in renal disease are summarized in Table 2.

Taken together, these toxins should not be viewed merely as isolated virulence determinants but as important amplifiers of disease severity within the unique microenvironment created by renal edema, immune dysfunction, and impaired tissue defense. Their biological effects become particularly relevant when host protective mechanisms are already compromised, as commonly observed in nephrotic syndrome, advanced CKD, and dialysis-dependent kidney disease.

### 4.3. Regulatory Mechanisms of Cutaneous Immune Evasion

The pathogenic program of *S. aureus* during skin infection is not constitutive but dynamically regulated in response to host signals. This regulatory flexibility may be particularly advantageous in patients with renal edema and kidney disease, where impaired neutrophil function, complement abnormalities, reduced opsonization capacity, and defective immune surveillance create a permissive environment for bacterial persistence. A landmark 2021 study by Kwiecinski et al. identified the ArlRS two-component regulatory system and its downstream effector MgrA as principal regulators of cutaneous immune evasion and bacterial survival during skin infection [24].

*S. aureus* strains lacking ArlRS or MgrA show dramatically reduced virulence in murine skin infection models: they fail to form the structured abscess architecture that normally sequesters bacteria from immune effectors, express lower levels of leukocidins, CHIPS (chemotaxis inhibitory protein), and SCIN (staphylococcal complement inhibitor), and are unable to kill neutrophils, block chemotaxis, degrade neutrophil extracellular traps, or survive direct neutrophil attack [24]. ArlRS and MgrA thus represent compelling therapeutic targets—pharmacological disruption of this regulatory cascade could simultaneously disable multiple immune evasion mechanisms, restoring susceptibility to innate immune clearance [25,26].

The potential relevance of the ArlRS/MgrA pathway may be even greater in patients with renal disease, where several innate immune defense mechanisms are already compromised by nephrotic and uremic immune dysfunction. In this setting, bacterial strategies that inhibit neutrophil recruitment, impair complement-mediated clearance, and promote survival within inflamed tissues may contribute disproportionately to persistent colonization, recurrent SSTIs, and progression toward invasive infection. Consequently, targeting regulatory systems such as ArlRS/MgrA may represent a particularly attractive therapeutic strategy for patients with kidney disease, in which restoration of host immune effectiveness could complement conventional antimicrobial therapy.

## 5. Clinical Implications

Translating the pathophysiological and virulence mechanisms described in Section 2 and Section 3 into clinical practice requires an understanding of how they manifest at the bedside. This section covers three interconnected clinical dimensions: the spectrum of SSTI presentations specifically observed in patients with renal oedema, and how the edematous tissue environment modifies the natural history of each (Section 5.1); the bidirectional relationship between staphylococcal bacteremia and acute kidney injury, and the vicious cycle this creates (Section 5.2); and the diagnostic challenges unique to edematous renal patients, where classical inflammatory signs may be masked and atypical presentations are common (Section 5.3).

### 5.1. Clinical Spectrum of S. aureus SSTIs in Renal Patients

The clinical presentations of *S. aureus* SSTIs in patients with renal edema span the full spectrum from superficial to life-threatening disease, but several features are particularly characteristic of this population. The edematous tissue environment lowers the inoculum required for infection, accelerates local spread by disrupting fascial barriers waterlogged with interstitial fluid, and favors the development of chronic, poorly healing wounds. Additionally, immunosuppressive agents commonly used in the management of nephrotic syndrome—corticosteroids, calcineurin inhibitors, and rituximab—further impair bactericidal defenses.

The pathogenic bacterium *S. aureus*, the most common pathogen isolated in SSTIs in the United States, presents with clinical features ranging from superficial infections with local symptoms to monomicrobial necrotizing fasciitis with systemic manifestations and potentially fatal outcomes [2]. In CKD patients, this spectrum is skewed toward more severe presentations, with a higher rate of bacteremic dissemination and multifocal disease.

The spectrum of cutaneous *S. aureus* infection in renal patients ranges from superficial localized disease to rapidly progressive necrotizing infection and sepsis. Edema-associated barrier disruption, lymphatic dysfunction, vascular access devices, and immunosuppressive therapy all contribute to distinct clinical risk profiles in this population. The major SSTI presentations and their associated renal-specific risk factors are outlined in Table 3.

### 5.2. The Bidirectional Relationship: Staphylococcal Infection and Acute Kidney Injury

The relationship between *S. aureus* and renal function is bidirectional: renal impairment predisposes to staphylococcal infection, while staphylococcal bacteremia (SAB) can precipitate or worsen acute kidney injury (AKI). AKI is a frequent and clinically significant complication of SAB, impacting management decisions and prognosis. A 2022 multicentre retrospective cohort study by Westgeest et al. [27] quantified the incidence of AKI within 14 days of SAB onset and evaluated its association with 30-day mortality. The study demonstrated that AKI occurred at a clinically meaningful frequency and was independently associated with adverse outcomes, establishing a mechanistic basis for the “staphylo-renal” vicious cycle: bacteremia triggers tubular injury and glomerulonephritis, worsening edema and immune dysfunction, which in turn promote further infection [27].

Mechanisms by which SAB induces AKI include: (1) haematogenous seeding of the renal parenchyma, with cortical abscesses or diffuse nephritis; (2) immune complex-mediated glomerulonephritis, driven by circulating staphylococcal superantigens and immune complexes depositing in glomerular capillaries; (3) haemodynamic insult from sepsis-associated vasodilation; and (4) direct nephrotoxicity from antibiotic therapy—particularly aminoglycosides and high-trough vancomycin—compounding the infective injury.

### 5.3. Diagnostic Considerations in the Renal Patient

Diagnosis of staphylococcal SSTIs in patients with renal edema presents specific clinical challenges. The classical inflammatory signs—erythema, warmth, and tenderness—may be attenuated or partially masked by edema, delaying recognition. Necrotizing infections in particular may be overlooked in the early stages, as the overlying skin can appear relatively normal while deep fascial necrosis progresses. A high index of suspicion, supported by imaging (magnetic resonance imaging (MRI) or computed tomography (CT) for deep infections) and early surgical exploration where indicated, is essential [15].

Microbiological sampling should include wound swabs or pus cultures from all accessible lesions, with blood cultures mandatory in the presence of systemic inflammatory signs. Susceptibility testing must include MRSA screening, given the high MRSA prevalence in healthcare-associated settings and dialysis populations. Nasal swabbing for MRSA colonization status should be performed in all hospitalized CKD patients, as colonization status directly guides empirical antibiotic selection and decolonization protocols [28,29,30].

Beyond local tissue injury, the clinical consequences of cutaneous *S. aureus* infection may be particularly severe in patients with kidney disease. Recurrent healthcare exposure, dialysis access devices, impaired immune defenses, delayed wound healing, and frequent antimicrobial use collectively increase the risk of recurrent SSTIs, bacteremia, metastatic infection, hospitalization, and treatment failure. Consequently, *S. aureus* infection should be viewed not merely as a cutaneous complication but as a clinically significant contributor to morbidity across CKD, dialysis-dependent, and nephrotic populations. These considerations underscore the need for therapeutic strategies specifically adapted to the pathophysiological and pharmacokinetic challenges encountered in patients with renal disease.

## 6. Therapeutic Perspectives

Management of staphylococcal SSTIs in patients with renal edema requires adapting standard antibiotic protocols to the pharmacokinetic and immunological constraints of renal disease. This section addresses five therapeutic domains in sequence: the evidence-based principles and guideline recommendations governing SSTI treatment stratified by severity and MRSA risk (Section 6.1); the comparative efficacy and renal dose adjustments of novel anti-MRSA agents approved in the past decade (Section 6.2); network meta-analysis evidence comparing these agents in MRSA infections (Section 6.3); the pharmacokinetic consequences of oedema on antibiotic distribution and clearance, and the implications for dosing (Section 6.4); and decolonisation strategies specifically validated in the nephrological setting (Section 6.5).

The management of cutaneous *S. aureus* infection in patients with renal disease extends beyond standard SSTI treatment algorithms. Renal dysfunction, edema-associated tissue alterations, immune dysregulation, dialysis-related factors, and substantial pharmacokinetic variability frequently influence both antimicrobial selection and treatment outcomes. Consequently, therapeutic strategies must be adapted to the unique pathophysiological characteristics of the renal host.

### 6.1. SSTI Management Principles in Patients with Renal Edema and Kidney Disease

Although the fundamental principles of SSTI management are similar across patient populations, several important considerations distinguish the management of cutaneous *S. aureus* infection in patients with renal edema and kidney disease. Altered antimicrobial pharmacokinetics, impaired immune function, higher rates of healthcare exposure, dialysis access devices, and increased susceptibility to recurrent infection frequently complicate otherwise standard treatment algorithms.

Antibiotic selection for *S. aureus* SSTIs must be guided by (1) the severity of infection, (2) the local and patient-specific MRSA risk, (3) the patient’s renal function, and (4) susceptibility data from cultures when available. The IDSA and UK guidelines both adopt a severity-stratified approach, distinguishing between non-purulent cellulitis (predominantly streptococcal), purulent infections (primarily staphylococcal), and severe/complicated SSTIs. For mild purulent SSTI (abscesses, furunculosis), incision and drainage (I&D) remains the cornerstone of management, with systemic antibiotics reserved for systemic signs, marked cellulitis, or immunocompromised hosts—including CKD patients on immunosuppression. For moderate-to-severe SSTI with systemic features, empirical antibiotic therapy targeting both MSSA and streptococci is appropriate, with escalation to anti-MRSA agents when risk factors are present [31,32,33,34].

For confirmed or strongly suspected MRSA SSTIs, UK guidelines recommend intravenous glycopeptides (vancomycin or teicoplanin) for severe cellulitis or soft-tissue infections; linezolid (oral or intravenous) or daptomycin (intravenous) as validated alternatives [31]. The IDSA guidelines (2011) recommend a first-generation cephalosporin or antistaphylococcal penicillin for MSSA, or vancomycin, linezolid, daptomycin, telavancin, or ceftaroline where MRSA risk factors are present [32].

Consequently, management decisions for renal patients should extend beyond conventional SSTI treatment principles to include assessment of renal function, edema severity, dialysis status, vascular access devices, antimicrobial dosing requirements, and the increased risk of treatment failure or recurrence. These disease-specific considerations form the basis for the therapeutic strategies discussed in the following sections.

### 6.2. Novel Anti-MRSA Agents: Evidence and Renal Considerations

Over the past decade, several new antibiotics with activity against MRSA have received regulatory approval for SSTIs, expanding the therapeutic armamentarium beyond vancomycin and linezolid. A 2023 in vitro study evaluated ceftobiprole, dalbavancin, tedizolid, and comparators against 124 clinical MRSA isolates from SSTIs (2020–2022), demonstrating that vancomycin remains preferred for complicated SSTIs, with linezolid and daptomycin as established alternatives, while newer agents offer additional options in specific clinical scenarios [35].

Selection of anti-MRSA therapy in patients with renal dysfunction requires balancing antimicrobial efficacy against the risk of nephrotoxicity, altered pharmacokinetics, dialysis-related clearance, and the feasibility of therapeutic drug monitoring. Several newer agents have expanded the available therapeutic options for complicated SSTIs, although renal-specific pharmacokinetic data remain limited for some compounds. The principal anti-MRSA agents, renal dose adjustments, and major clinical considerations are summarized in Table 4.

### 6.3. Comparative Efficacy of Anti-MRSA Agents

A network meta-analysis published in 2024 evaluated the efficacy and safety of multiple antibiotics for MRSA infections. The analysis compared clinical cure and microbiological eradication rates, as well as adverse event profiles, across subgroups, including complex SSTIs (cSSSIs and cSSTIs) and pneumonia [36]. While vancomycin remains the benchmark comparator, the analysis identified several agents—including linezolid, tedizolid, and daptomycin—with non-inferior or superior clinical cure rates in specific subgroup analyses, providing evidence-based guidance for individual patient selection.

For persistent or refractory MRSA bacteremia—a scenario encountered in dialysis patients with vascular access infections—the combination of daptomycin plus ceftaroline has been studied as salvage therapy. A 2023 retrospective study evaluated outcomes of daptomycin + ceftaroline versus alternative therapy in patients with persistent MRSA bacteremia, stratified by baseline renal function (creatinine clearance and renal replacement therapy status). The combination demonstrated clinical benefit in treatment-refractory cases, though its use in patients with edematous CKD requires careful pharmacokinetic monitoring [37].

### 6.4. Pharmacokinetic Considerations in Renal Edema

Edematous states substantially alter antibiotic pharmacokinetics, increasing the risk of therapeutic failure due to underdosing or toxicity from drug accumulation. Increased volume of distribution, driven by expanded interstitial and total body water, lowers peak serum concentrations of hydrophilic antibiotics (vancomycin, beta-lactams, aminoglycosides), requiring higher loading doses to achieve therapeutic levels. Conversely, impaired renal clearance prolongs the half-life of renally excreted antibiotics, necessitating dose reduction or interval extension to avoid accumulation.

Multidisciplinary algorithms for targeted antimicrobial therapy of severe *S. aureus* infections, published in 2023, organize antibiotic selection by site of infection and use PK/PD parameters to inform therapeutic decision-making [38]. For patients on renal replacement therapy (RRT), including intermittent hemodialysis (IHD) and continuous renal replacement therapy (CRRT), the pharmacokinetic profiles of virtually all anti-MRSA agents are further altered, with drug clearance influenced by dialysis modality, membrane type, and ultrafiltration rate. Specialized dosing guidelines for RRT patients are referenced in institutional antimicrobial stewardship resources.

### 6.5. Decolonization Strategies in the Nephrological Setting

Decolonization—the systematic eradication of *S. aureus* nasal and skin carriage—is a well-established strategy for reducing the risk of subsequent invasive infection in high-risk populations. In nephrology, decolonization is particularly relevant for patients undergoing peritoneal dialysis (PD) and HD with arteriovenous fistulae or central venous catheters, where staphylococcal access-site infections carry substantial morbidity.

A systematic review and meta-analysis of 9 studies by Grothe et al. [28], comprising 839 PD patients colonized with *S. aureus*, found that both topical mupirocin and systemic antibiotic decolonization significantly reduced the incidence of access-site infections compared with placebo or no treatment. Mupirocin 2% nasal ointment, applied to the anterior nares twice daily for 5 days (with or without skin decolonization using chlorhexidine), remains the standard first-line approach [28]. An important caveat is the emergence of mupirocin resistance, which has reached clinically significant levels in some settings. For patients colonized with mupirocin-resistant MRSA (MupR-MRSA), alternative decolonization regimens—including retapamulin, povidone-iodine, or systemic rifampicin—may be considered, though evidence remains limited and should be guided by local susceptibility patterns.

## 7. Management of *S. aureus* Infections in the Intensive Care Unit (ICU)

The intensive care unit represents the most challenging arena for managing *S. aureus* infections in the renal patient, where the compounding of critical illness, organ failure, and invasive monitoring creates a uniquely hostile pharmacological and immunological environment. This section addresses four interrelated aspects of ICU management: the epidemiology and clinical presentations of staphylococcal infections in the critically ill renal patient, with their associated mortality burden (Section 7.1); the profound pharmacokinetic alterations—augmented renal clearance, expanded volume of distribution, dialysis-dependent clearance—that render standard dosing unreliable in this population (Section 7.2); the evidence base for AUC/MIC-guided vancomycin dosing as the current pharmacodynamic standard of care in the ICU (Section 7.3); and the integrated principles of empirical antibiotic strategy, mandatory source control, and antimicrobial stewardship specific to the ICU context (Section 7.4).

### 7.1. Epidemiology and Clinical Significance in the ICU

*S. aureus*—and in particular MRSA—is one of the leading causative agents of severe infections in the intensive care unit, accounting for a disproportionate share of ICU-acquired bacteremia, ventilator-associated pneumonia, catheter-related bloodstream infections, and complicated SSTIs. The critically ill patient with renal edema represents a convergence of the most adverse prognostic factors: immune dysfunction, vascular access requirements, disrupted skin barriers, and a pharmacokinetic environment radically different from that of the non-ICU patient.

Evidence-based algorithms for targeted antibiotic therapy of severe *S. aureus* infections in critically ill adult patients have been developed through multidisciplinary expert consensus, incorporating a review and organizing therapeutic choices by site of infection and PK/PD parameters—distinguishing between MSSA and MRSA, covering endocarditis, primary bacteremia, intravascular device infections, community-acquired pneumonia, CNS infections, and necrotizing skin and soft tissue infections [38].

The critically ill renal patient faces a specific and compounding threat: sepsis-associated kidney injury is common in critically ill patients and significantly increases morbidity and mortality, with several complex pathophysiological factors contributing—including macrocirculatory and microcirculatory changes, mitochondrial dysfunction, and metabolic reprogramming [39]. When *S. aureus* is the precipitating pathogen, the renal injury both compromises drug clearance and amplifies the immunological vulnerability that drove the infection in the first place, creating a vicious cycle of escalating severity.

A striking illustration is provided by a 2025 case report of a steroid-treated patient with end-stage renal disease who developed fulminant PVL-positive MSSA pneumonia requiring venovenous extracorporeal membrane oxygenation and continuous haemodiafiltration. Severe hyperkalemia, metabolic acidosis, and lactic acidosis complicated the course, with persistent hemodynamic compromise despite maximal ICU support—illustrating the lethal convergence of staphylococcal virulence and end-stage renal disease in the critical care setting [40].

In critically ill renal patients, *S. aureus* infection may present through multiple overlapping syndromes, including catheter-related bloodstream infection, ventilator-associated pneumonia, necrotizing SSTI, and septic shock with secondary organ dysfunction. Early recognition of the primary infectious focus and prompt diagnostic evaluation are essential because mortality rises substantially once bacteremia and multiorgan failure develop. The principal ICU presentations, diagnostic priorities, and associated mortality impact are summarized in Table 5.

### 7.2. Pharmacokinetic Alterations in the ICU Renal Patient

The pharmacokinetics of antibiotics—and of vancomycin in particular—are profoundly altered in the critically ill patient, making standard dosing regimens unreliable. Two opposing physiological extremes must be recognized and actively managed.

Augmented renal clearance (ARC) occurs paradoxically in a subset of ICU patients—particularly younger, hyperdynamic septic patients—where glomerular filtration is supranormal (eGFR > 130 mL/min/1.73 m^2^). ARC is not rare in the ICU—estimates suggest it affects 30–65% of young critically ill patients—and represents a pharmacokinetic emergency requiring active recognition [41].

Conversely, established CKD and renal edema produce the opposite problem: drug accumulation through impaired clearance, risking nephrotoxicity from the very agents needed to treat the infection. Critically ill patients admitted to the ICU may have markedly altered pharmacokinetic parameters compared to non-critically ill patients; individualized dose adjustment and therapeutic drug monitoring of vancomycin are therefore essential. A 24-h AUC target of 700 µg·h/mL has been proposed as a more reliable pharmacokinetic parameter to achieve sufficient clinical efficacy while preventing vancomycin-induced nephrotoxicity in this population [42].

Critically ill patients on extracorporeal organ support, CRRT, IHD, or ECMO pose an additional pharmacokinetic challenge: drug clearance depends on membrane type, surface area, ultrafiltration rate, and dialysate flow, rendering standard dosing nomograms unreliable [43]. Liaison with clinical pharmacology and infectious disease is mandatory for these patients.

### 7.3. AUC-Guided Vancomycin Dosing in the ICU

The 2020 revised consensus guidelines from ASHP/IDSA/SIDP marked a paradigm shift in vancomycin monitoring: from trough-based to AUC/MIC-based dosing. The guidelines recommend a loading dose of 15–20 mg/kg followed by maintenance dosing targeting an AUC/MIC of 400–600 mg·h/L (based on an assumed MRSA MIC of 1 mg/L), with AUC/MIC estimated by multiplying the vancomycin steady-state concentration by 24 to approximate AUC_0–24_ [44].

For MRSA bacteremia, multivariate analyses have confirmed that lower initial AUC/MIC is a significant independent risk factor for treatment failure. Current IDSA recommendations use AUC as the primary pharmacodynamic monitoring indicator before formal MIC data become available [45]. A multicentre retrospective Japanese ICU study (2020–2022) quantified nephrotoxicity risk: the data indicate that initial dosing should target an AUC on day 2 not exceeding 500 µg·h/mL in patients at high risk of AKI—a critical threshold for patients at risk of renal edema [46].

Monte Carlo simulations comparing vancomycin infusion modes in critically ill MRSA patients demonstrated that the probability of target attainment (PTA) at AUC_0–24_/MIC 400–600 varies significantly by renal function stratum, with continuous infusion and optimized two-step infusion offering PK/PD advantages over standard intermittent infusion in patients with altered renal pharmacokinetics [47].

Antibiotic management of severe *S. aureus* infection in critically ill renal patients requires continuous integration of infection severity, renal function, extracorporeal support modalities, and pharmacokinetic/pharmacodynamic targets. Expanded volume of distribution, augmented renal clearance, and dialysis-dependent drug removal frequently necessitate individualized dosing strategies and therapeutic drug monitoring. The major ICU anti-staphylococcal agents and their pharmacokinetic considerations in renal impairment are summarized in Table 6.

### 7.4. Empirical Strategy, Source Control, and Antimicrobial Stewardship

In the ICU patient with suspected *S. aureus* sepsis and renal edema, empirical antibiotic selection must simultaneously address four imperatives: (1) adequate MRSA coverage initiated within one hour of sepsis recognition; (2) appropriate loading doses to account for expanded volume of distribution; (3) avoidance of further nephrotoxic insult; and (4) daily reassessment with de-escalation as culture data become available. Dual coverage for MRSA should be considered for high-risk ICU patients; an initial loading-dose strategy is essential to rapidly attain the target, with subsequent dosing guided by renal function and infectious disease consultation [48].

Antibiotic therapy alone is insufficient in ICU *S. aureus* infections associated with identifiable foci. Source control—surgical debridement of necrotizing SSTIs, removal of infected vascular catheters, and drainage of deep abscesses—is a prerequisite for treatment success. Management of *S. aureus* bloodstream infections in critically ill patients requires a structured multidisciplinary approach integrating infectious disease consultation, echocardiography to exclude infective endocarditis, and repeated blood cultures to document bacteremia clearance—with persistent bacteremia beyond 72 h triggering investigation for metastatic foci including renal cortical abscesses, osteomyelitis, and septic arthritis [49].

Antimicrobial stewardship in the ICU context demands: daily antibiotic reassessment (“antibiotic time-out”); de-escalation from broad-spectrum empirical therapy to targeted agents as soon as susceptibility data permit; avoidance of combination therapy beyond the initial empirical phase unless specifically indicated (e.g., daptomycin + ceftaroline for persistent MRSA bacteraemia); and rigorous monitoring for antibiotic-associated nephrotoxicity—particularly in patients already burdened by renal oedema and CKD.

**Figure 4:**
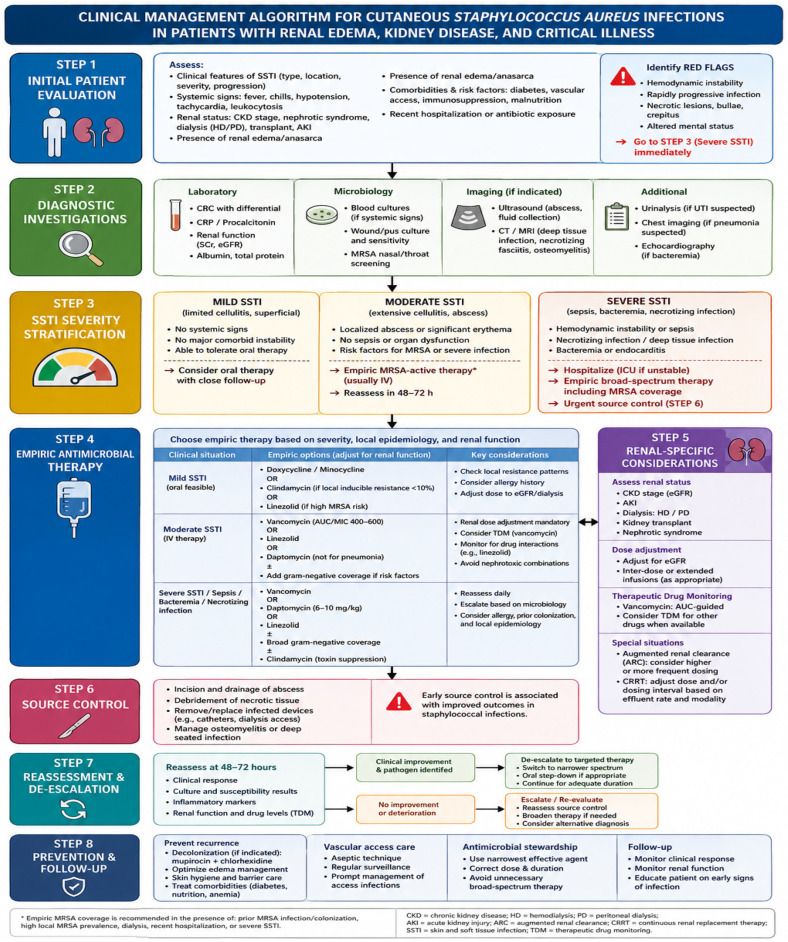
Clinical management algorithm for suspected or confirmed cutaneous *S. aureus* infections in patients with renal edema, kidney disease, and critical illness (Figure created in Canva, https://www.canva.com; accessed on 9 June 2026). The algorithm summarizes a practical diagnostic and therapeutic approach integrating initial clinical evaluation, microbiological investigations, SSTI severity stratification, empiric and targeted antimicrobial therapy, renal-specific dose adjustment, therapeutic drug monitoring, source-control interventions, and follow-up management. Special considerations relevant to chronic kidney disease, dialysis, augmented renal clearance, continuous renal replacement therapy, and intensive care unit settings are highlighted to facilitate individualized clinical decision-making. Black arrows indicate the recommended progression through the diagnostic and therapeutic algorithm. Branching arrows represent alternative clinical pathways determined by SSTI severity, microbiological results, renal function, and treatment response.

Given the complexity of managing cutaneous *Staphylococcus aureus* infections in patients with renal edema, kidney disease, and critical illness, a structured diagnostic and therapeutic approach is essential. The integration of SSTI severity assessment, microbiological investigations, renal-specific pharmacokinetic considerations, antimicrobial selection, therapeutic drug monitoring, and source-control measures is summarized in Figure 4.

## 8. Discussion

### 8.1. Principal Findings and Their Synthesis

The central thesis of this review is that renal edema represents a biologically active risk factor for cutaneous *S. aureus* infection through three interconnected mechanisms: disruption of the skin barrier, nephrotic and uremic immune dysfunction, and impaired lymphatic immune surveillance. Experimental evidence supporting the role of ArlRS/MgrA in immune evasion and abscess formation, together with studies demonstrating the effects of lymphatic dysfunction on cutaneous inflammation, provides mechanistic support for the increased SSTI burden observed in CKD and dialysis populations [11,15,24].

A major contribution of this review is the integration of these mechanisms with renal-specific pharmacokinetic challenges encountered in critical care, including augmented renal clearance, expanded volume of distribution, and extracorporeal drug removal. The reported failure of maximum-dose vancomycin in a patient with ARC illustrates how altered pharmacokinetics may compromise effective MRSA therapy despite guideline-based treatment [50].

Although renal edema is the primary focus, these mechanisms likely extend across a broader spectrum of kidney disease, including CKD, dialysis, and critical illness, where immune dysfunction, tissue vulnerability, and altered antimicrobial exposure interact to increase susceptibility to staphylococcal infection.

### 8.2. Controversies and Unresolved Questions

Several important questions remain unresolved. The relative contributions of the underfill and overfill mechanisms in nephrotic edema vary among patients and may influence the degree of immune dysfunction [5]. Likewise, although PVL-positive strains have been associated with severe SSTIs, experimental findings remain inconsistent, and their specific contribution in immunocompromised renal patients has not been prospectively established [20,21].

The evidence supporting MRSA decolonization in CKD is largely derived from dialysis populations, and the increasing prevalence of mupirocin resistance further limits generalizability [28]. Moreover, although CKD and nephrotic syndrome are consistently associated with increased rates of colonization, SSTIs, bacteremia, and infection-related hospitalization, most studies do not distinguish the independent contribution of edema from other risk factors such as diabetes, vascular access devices, immunosuppression, malnutrition, and healthcare exposure [51,52]. Consequently, renal edema should be considered one component of a multifactorial susceptibility profile rather than the sole determinant of infection risk.

### 8.3. Antimicrobial Resistance: The Overarching Threat

The clinical challenges described throughout this review must be contextualized within the global AMR crisis. A 2024 systematic analysis with forecasts to 2050 estimated that MRSA represented the pathogen-drug combination responsible for both the largest increase in attributable AMR burden from 1990 to 2021 and the largest absolute attributable mortality in 2021, across five WHO super-regions—despite being classified as “high” rather than “critical” priority due to its treatability profile. Forecasts project 1.91 million deaths attributable to AMR annually by 2050, with MRSA remaining a significant contributor [53].

For renal patients specifically, the AMR burden is compounded by the structural conditions of nephrological care: high antibiotic exposure over years of chronic disease; repeated hospitalizations; dialysis access, which provides a persistent portal for healthcare-associated MRSA acquisition; and immunosuppressive therapies that reduce the selective pressure required to contain resistant clones. The mortality rate from MRSA infection in hemodialysis patients is reported to be five times higher than in hemodialysis patients without MRSA infection—a stark illustration of the intersection between renal vulnerability and antimicrobial resistance [54].

### 8.4. Emerging Therapeutic Strategies: Anti-Virulence and Immunomodulatory Approaches

As summarized in Table 7, most anti-virulence strategies remain at the preclinical or early translational stage, with conventional anti-MRSA antibiotics remaining the only established therapeutic option. Nevertheless, approaches targeting toxin activity, quorum sensing, and immune evasion may eventually complement conventional antimicrobial therapy, particularly in immunocompromised renal patients.

Among these, anti-α-toxin monoclonal antibodies currently represent the most advanced strategy. Experimental studies have demonstrated protection against invasive *S. aureus* infection, while bispecific antibodies targeting both α-toxin and ClfA have shown broader efficacy in preclinical models [55,56]. Quorum-sensing inhibition remains promising but may paradoxically enhance biofilm persistence in chronic infections, limiting its applicability in CKD patients with biofilm-associated device infections [57].

Overall, these approaches remain investigational, and prospective clinical trials are required before routine implementation in patients with renal disease.

### 8.5. Strength of the Current Evidence

Although several mechanistic pathways discussed in this review are supported by direct observations in nephrotic syndrome, chronic kidney disease, and dialysis populations, other proposed mechanisms are derived from biologically analogous conditions, including chronic edema, lymphedema, and critical illness. Consequently, the strength of evidence is not uniform across all pathways. While the associations between renal-associated immune dysfunction and susceptibility to infection are supported by substantial clinical and experimental evidence, the specific contributions of edema-related lymphatic impairment and tissue microenvironmental changes remain less directly established. These limitations highlight the need for dedicated prospective studies specifically evaluating the relationship between renal edema and cutaneous *S. aureus* infection.

Additional limitations arise from the predominance of observational studies, retrospective analyses, and mechanistic investigations. Many available studies were not specifically designed to evaluate the risk of edema-associated SSTI, limiting the ability to establish causality. Furthermore, substantial heterogeneity exists regarding patient populations, definitions of infection, severity of renal disease, and adjustment for confounding variables. These methodological differences complicate direct comparisons across studies and may contribute to inconsistencies in the reported associations.

To improve transparency regarding the evidentiary basis of the proposed pathophysiological framework, Table 8 summarizes the principal mechanisms discussed in this review and categorizes them by source and overall strength of supporting evidence.

### 8.6. Limitations of the Present Review

Several methodological limitations of this narrative review must be acknowledged. First, the intersection of renal edema and cutaneous staphylococcal infection has not been the subject of dedicated prospective clinical trials; the pathophysiological links described are synthesized from mechanistic studies, observational cohorts, and clinical series that were not designed to address this specific intersection. Causal inference from such heterogeneous data is inherently limited.

Second, the populations studied in the cited literature are heterogeneous: nephrotic syndrome, CKD stages 3–5, ESRD on HD, and peritoneal dialysis patients differ substantially in their immune phenotype, pharmacokinetics, and risk of MRSA exposure. Generalization across these subgroups requires caution.

Third, while the review covers literature through December 2025, rapidly evolving areas—particularly anti-virulence therapeutics, Bayesian pharmacokinetic approaches for AUC-guided vancomycin dosing, and molecular diagnostics—may have developed significantly since the cited studies were published. Clinicians should consult current guideline updates and institutional antimicrobial stewardship resources for the most up-to-date recommendations.

A further limitation is that relatively few studies have specifically evaluated edema-associated staphylococcal infection as a distinct clinical entity. Consequently, some mechanistic concepts discussed in this review are extrapolated from broader populations, including CKD, nephrotic syndrome, dialysis, and critical care. Nevertheless, these conditions share many of the immunological, vascular, and pharmacokinetic abnormalities that constitute the conceptual framework proposed herein.

### 8.7. Research Agenda

The following research priorities emerge from the synthesis and critical appraisal above:

(1) Prospective cohort studies specifically designed to characterize the incidence, clinical spectrum, and outcomes of *S. aureus* SSTIs in patients with nephrotic syndrome and CKD, stratified by edema severity, immunosuppressive burden, and MRSA carriage status.
(2) Pharmacokinetic studies of novel anti-MRSA agents (tedizolid, dalbavancin, oritavancin) in oedematous CKD and ICU patients, specifically addressing the impact of expanded volume of distribution and dialysis modality on target attainment.
(3) Randomized controlled trials of decolonization regimens for mupirocin-resistant MRSA in dialysis populations, evaluating alternatives including retapamulin, povidone-iodine, and oral rifampicin combinations.
(4) Translational studies evaluating ArlRS/MgrA inhibitors in murine models of edema-associated staphylococcal skin infection, specifically in the context of uremic immune dysfunction.
(5) Biomarker discovery and validation studies aimed at identifying readily measurable serum, urine, inflammatory, and microbiome-related biomarkers (e.g., uremic toxin profiles, complement factor levels, neutrophil function assays, cytokine signatures, and microbiome-derived metabolites) capable of predicting SSTI risk, disease severity, recurrence, and response to therapy in patients with CKD and renal edema. Such biomarkers could facilitate individualized risk stratification and support targeted prevention and decolonization strategies.
(6) Microbiome-centered investigations exploring the interplay between gut dysbiosis, cutaneous microbial ecology, uremic toxin generation, and susceptibility to *S. aureus* colonization and infection in CKD and dialysis populations. Particular attention should be directed toward the gut–skin axis as a potential contributor to immune dysregulation and impaired cutaneous host defense.
(7) Prospective longitudinal studies evaluating edema severity as an independent predictor of SSTI incidence, recurrence, bacteremia, and infection-related hospitalization. Such investigations should incorporate standardized edema assessment tools and adjust for major confounders, including diabetes mellitus, immunosuppressive therapy, nutritional status, vascular access devices, and healthcare exposure.

## 9. Conclusions

Renal edema represents far more than a passive manifestation of fluid overload. The combined effects of barrier disruption, nephrotic and uremic immune dysfunction, impaired lymphatic surveillance, and altered antimicrobial pharmacokinetics create a biologically permissive microenvironment for cutaneous *S. aureus* infection. In this setting, *S. aureus* can exploit structural skin fragility, diminished innate and adaptive immune responses, and compromised tissue defenses to establish persistent colonization, recurrent SSTIs, and invasive disease. The interaction between renal-associated host vulnerability and bacterial virulence is particularly relevant in patients with CKD, nephrotic syndrome, dialysis-dependent kidney disease, and critical illness. Recognition of renal edema as a clinically relevant modifier of host–pathogen interactions may facilitate more individualized prevention and treatment strategies across CKD, dialysis, and critically ill renal populations. In addition, expanded volume of distribution, augmented renal clearance, extracorporeal renal support, and fluctuating renal function substantially complicate antimicrobial therapy, emphasizing the need for renal-adapted dosing strategies, therapeutic drug monitoring, source control, infection prevention, and multidisciplinary care. Beyond conventional antibiotic therapy, emerging anti-virulence and immunomodulatory approaches may offer important future therapeutic opportunities. Strategies targeting toxin activity, quorum-sensing systems, biofilm regulation, and immune evasion pathways may complement existing antimicrobial therapies while limiting selective pressure for resistance. Given the growing global burden of MRSA and the rising prevalence of advanced renal disease, further translational and clinical research is needed to better define the mechanisms, prevention strategies, and optimal management of edema-associated staphylococcal infection in patients with renal disease.

## Figures and Tables

**Table 1:** Mechanisms of nephrotic immunodeficiency and their clinical consequences.

Mechanism	Pathophysiological Basis	Clinical Consequence
Urinary loss of immunoglobulins	Massive proteinuria includes IgG loss; Reduced opsonization capacity	Increased susceptibility to encapsulated and extracellular bacteria, including *S. aureus*
T-cell dysfunction	Th2/Th1 imbalance; reduced Treg function; increased Th17 activity	Impaired cell-mediated immunity; Recurrent and persistent infections
B-cell dysregulation	Increased memory B-cell activity; altered CD23 release; anti-CD40 autoantibodies	Dysregulated antibody production; Impaired humoral immune response
Complement loss	Urinary loss of complement factors (Factor B, Factor D, Properdin)	Defective opsonophagocytosis; Increased bacterial survival
Corticosteroid therapy	Immunosuppressive treatment used in NS management	Iatrogenic immunosuppression compounding nephrotic immunodeficiency
Hypoalbuminaemia	Reduced serum albumin as acute-phase protein carrier	Reduced drug and antimicrobial peptide transport; nutritional depletion

**Table 2:** Key *S. aureus* virulence factors, mechanisms, and relevance in patients with renal disease.

Toxin/Factor	Mechanism	Predominant Clinical Manifestation	Relevance in Renal Patients
Alpha-toxin (Hla)	Pore formation in epithelial/endothelial membranes	Furuncles, wound infections, cellulitis	Enhanced effect in hypoalbuminemia; reduced opsonization
PVL (LukS/F-PV)	Pore formation in neutrophils, monocytes, and macrophages	Necrotizing furunculosis, recurrent abscesses, CA-MRSA SSTI	PMN dysfunction in CKD amplifies PVL-mediated immune evasion
Exfoliative toxins (ETA, ETB)	Desmoglein-1 cleavage; intraepidermal cleavage	Bullous impetigo, SSSS	Renal clearance-dependent; toxic accumulation in CKD/AKI
TSST-1 (Superantigen)	Non-specific T-cell activation; cytokine storm	Toxic shock syndrome, multiorgan failure	Exaggerated systemic response in immunocompromised CKD patients
IsdA/SpA (Protein A)	Fc binding; complement evasion; iron sequestration	Persistent bacteremia, biofilm formation	Biofilm formation on skin and vascular access sites in dialysis patients
ArlRS/MgrA (Regulatory cascade)	Controls the expression of immune evasion factors (leukocidins, SCIN, CHIPS)	Abscess formation; neutrophil evasion	Therapeutic target; dysregulated in high-inoculum CKD infections

**Table 3:** Clinical spectrum of *S. aureus* SSTIs and associated risk factors in patients with renal edema.

Clinical Presentation	Key Features	Typical Organism	Risk Factors in CKD/Edema
Impetigo/ Ecthyma	Superficial erosions; honey-coloured crusting; ecthyma penetrates the dermis	*S. aureus* (MSSA/MRSA), GAS	Skin maceration from edema; poor hygiene; hypoalbuminemia
Folliculitis/ Furunculosis	Perifollicular pustules; nodular abscesses; frequent recurrence with PVL strains	*S. aureus* (PVL-positive)	Increased nasal carriage; immunosuppression; skin moisture
Cellulitis	Non-purulent spreading erythema, warmth, edema; lymphangitis possible	*S. aureus*, GAS	Pre-existing lymphatic dysfunction; skin barrier disruption
Erysipelas	Raised, sharply demarcated erythema; predominantly superficial lymphatics	GAS >> *S. aureus*	Lymphoedema; renal edema; Repeated episodes worsen lymphatics
Wound/ Access-site Infection	Purulent discharge; dehiscence; biofilm on catheter/fistula	*S. aureus*, MRSA, CONS	Dialysis access; peritoneal catheter; Reduced skin immunity
Necrotizing Fasciitis (Type II)	Rapid spread; systemic toxicity; dishwasher exudate; pain disproportionate to appearance	*S. aureus* (monomicrobial)	High mortality; delayed diagnosis in edematous limbs

**Table 4:** Anti-MRSA antibiotics for SSTIs: mechanisms, renal dose adjustments, and clinical considerations.

Antibiotic	Class	MRSA Activity	Dose Adjustment in CKD	Key Notes
Vancomycin	Glycopeptide	MSSA/ MRSA first-line	Required (AUC/MIC monitoring); CrCl < 50 mL/min requires dose reduction	Nephrotoxic; avoid high troughs in CKD; AUC-guided dosing preferred
Teicoplanin	Glycopeptide	MRSA first-line	Required; loading doses followed by dose/interval adjustment	Less nephrotoxic than vancomycin; once-daily dosing possible
Linezolid	Oxazolidinone	MRSA/ MSSA	No renal dose adjustment required	Oral bioavailability ~100%; risk of serotonin syndrome; myelosuppression with prolonged use
Daptomycin	Cyclic lipopeptide	MRSA/ MSSA	Required (CrCl < 30 mL/min: q48 h dosing)	Inactivated by pulmonary surfactant; CPK monitoring required; NOT for pneumonia
Ceftaroline	5th-gen cephalosporin	MRSA/ MSSA	Required (CrCl < 50 mL/min: dose reduction)	Only beta-lactam approved for MRSA; useful in vancomycin-intolerant patients
Tedizolid	Oxazolidinone (2nd-gen)	MRSA/ MSSA	No renal dose adjustment required	Once-daily; shorter course (6 days) vs. linezolid; less myelosuppression
Dalbavancin	Lipoglycopeptide	MRSA/ MSSA	Required (single 1500 mg dose; adjust if CrCl < 30)	Once-weekly or single-dose IV; useful for outpatient SSTI completion therapy
Oritavancin	Lipoglycopeptide	MRSA/ MSSA	Limited data in severe CKD	Single-dose IV; long half-life (245 h); limited renal CKD data

**Table 5:** Clinical presentations of *S. aureus* infection in the ICU renal patient, with diagnostic priorities and mortality impact.

ICU Presentation	Typical Source	MRSA Risk	Key Diagnostic Step	Mortality Impact
Catheter-related BSI (CRBSI)	CVC/dialysis catheter	High (≥40% in dialysis)	Blood cultures × 2 + catheter tip culture	30-day mortality 20–30%
Ventilator-associated pneumonia (VAP)	Endotracheal tube biofilm	High in post-influenza, immunosuppressed	BAL/protected brush + quantitative culture	Attributable mortality 10–15%
Necrotising SSTI → sepsis	Oedematous skin fissure/wound	Variable; PVL strains are more common in CA	CT/MRI + surgical exploration	Mortality 25–35% in renal patients
Haematogenous seeding	Primary bacteremia (CRBSI, SSTI)	MRSA bacteremia → metastatic foci	Echocardiography (IE exclusion); repeat BCs	Infective endocarditis: 30% mortality
Septic shock with AKI	Bacteremia + toxin-mediated vasodilation	MRSA/MSSA both; TSST-1 strains	SOFA score, lactate, organ function panel	>50% mortality in MRSA septic shock + CKD

ICU, intensive care unit; MRSA, methicillin-resistant Staphylococcus aureus; MSSA, methicillin-susceptible Staphylococcus aureus; BSI, bloodstream infection; CRBSI, catheter-related bloodstream infection; CVC, central venous catheter; BAL, bronchoalveolar lavage; PVL, Panton–Valentine leukocidin; CA, community-associated; CT, computed tomography; MRI, magnetic resonance imaging; IE, infective endocarditis; BCs, blood cultures; SOFA, Sequential Organ Failure Assessment; AKI, acute kidney injury; TSST-1, toxic shock syndrome toxin-1. The arrow (→) denotes progression from the primary infectious process or microbiological finding to a subsequent clinical complication or disease stage.

**Table 6:** Antibiotic management of *S. aureus*/MRSA infections in critically ill ICU patients with renal impairment.

Antibiotic	ICU Indication	Dosing in CKD/Edema	Key PK/PD Consideration	Avoid/Caution
Vancomycin IV	MRSA BSI, VAP, cSSTI—first line	AUC/MIC 400–600; loading 25–30 mg/kg in edema; TDM mandatory	Expanded Vd in edema → higher loading dose; ARC → rapid clearance	Nephrotoxic; AUC > 500 on day 2 → ↑ AKI risk
Daptomycin IV	MRSA BSI, right-sided endocarditis, SSTI	6–10 mg/kg q24 h; q48 h if CrCl < 30; CPK monitoring	Inactivated by the pulmonary surfactant—do NOT use for pneumonia/VAP	Monitor CPK weekly; rhabdomyolysis risk
Ceftaroline IV	MRSA BSI salvage, SSTI, pneumonia	600 mg q8 h in normal renal function; adjust by CrCl	Only beta-lactam approved for MRSA; useful combination with daptomycin in persistent MRSA bacteremia	Limited data in ESRD; pharmacist-guided dosing required
Linezolid IV/PO	MRSA VAP, SSTI, step-down therapy	600 mg q12 h—no renal dose adjustment required	Tissue penetration superior to vancomycin for lung/SSTI; 100% oral bioavailability	Serotonin syndrome; myelosuppression > 14 days; avoid with SSRIs
Teicoplanin IV	MRSA BSI, SSTI—alternative to vancomycin	Loading 6 mg/kg q12 h × 3, then q24–48 h by CrCl	Less nephrotoxic than vancomycin; once-daily maintenance; TDM target trough >15–20 mg/L	Slower bactericidal activity vs. vancomycin
Daptomycin + Ceftaroline	Persistent MRSA bacteremia (salvage)	Dose adjust both by renal function; pharmacist-guided	Synergistic: ceftaroline restores daptomycin susceptibility in daptomycin-tolerant strains	High cost; reserve for refractory cases

ICU, intensive care unit; MRSA, methicillin-resistant Staphylococcus aureus; MSSA, methicillin-susceptible *Staphylococcus aureus*; BSI, bloodstream infection; VAP, ventilator-associated pneumonia; cSSTI, complicated skin and soft tissue infection; CKD, chronic kidney disease; CrCl, creatinine clearance; TDM, therapeutic drug monitoring; Vd, volume of distribution; ARC, augmented renal clearance; PK/PD, pharmacokinetic/pharmacodynamic; CPK, creatine phosphokinase; ESRD, end-stage renal disease; IV, intravenous; PO, oral. The arrow (→) indicates a resulting pharmacokinetic or clinical consequence, whereas the upward arrow (↑) denotes an increased risk or level of the indicated parameter.

**Table 7:** Developmental Status of Emerging Anti-Virulence and Immunomodulatory Strategies Against *Staphylococcus aureus*.

Strategy	Representative Example	Development Stage	Current Clinical Applicability
Anti-alpha toxin monoclonal antibodies	YG1, MEDI4893-derived approaches	Preclinical/Early Translational	Not available for routine clinical use
Bispecific monoclonal antibodies	Anti-Hla + Anti-ClfA constructs	Preclinical	Not available for clinical use
Quorum-sensing inhibitors	Agr-targeted inhibitors	Preclinical	Experimental
ArlRS/MgrA inhibitors	Small-molecule regulatory inhibitors	Preclinical	Experimental
Immune-modulating anti-virulence strategies	Host-directed adjunctive therapies	Early translational	Investigational
Conventional anti-MRSA therapies	Vancomycin, Linezolid, Daptomycin, Ceftaroline	Established clinical use	Currently available

**Table 8:** Strength of Evidence Supporting the Proposed Pathophysiological Mechanisms Linking Renal Edema and Cutaneous *Staphylococcus aureus* Infection.

Proposed Mechanism	Direct Evidence in Renal Disease	Evidence from Related Conditions	Overall Strength of Evidence
Skin barrier disruption and fissuring	Nephrotic syndrome, CKD- associated edema	Chronic edema literature	Moderate–Strong
Urinary immunoglobulin loss and complement depletion	Nephrotic syndrome studies	—	Strong
Uremic immune dysfunction	CKD and dialysis studies	ICU immune dysfunction	Strong
PBURS-mediated neutrophil dysfunction	CKD studies	—	Strong
Lymphatic dysfunction and impaired immune surveillance	Limited renal-specific evidence	Lymphedema and chronic edema studies	Moderate
Biofilm persistence in edematous tissues	Indirect evidence	Chronic wound literature	Moderate
Altered antimicrobial pharmacokinetics	CKD, dialysis, ICU studies	—	Strong

## Data Availability

No new data were created or analyzed in this study. Data sharing is not applicable to this article.

## References

[1] Adeiza S.S., Islam A., Shittu A. (2024). Global, regional, and national burdens: An overlapping meta-analysis on *Staphylococcus aureus* and its drug-resistant strains. One Health Bull..

[2] Linz M.S., Mattappallil A., Finkel D., Parker D. (2023). Clinical Impact of *Staphylococcus aureus* Skin and Soft Tissue Infections. Antibiotics.

[3] Navidifar T., Zare Banadkouki A., Parvizi E., Mofid M., Golab N., Beig M., Sholeh M. (2025). Global prevalence of macrolide-resistant *Staphylococcus* spp.: A comprehensive systematic review and meta-analysis. Front. Microbiol..

[4] Syed-Ahmed M., Narayanan M. (2019). Immune Dysfunction and Risk of Infection in Chronic Kidney Disease. Adv. Chronic Kidney Dis..

[5] Frățilă V.-G., Lupușoru G., Sorohan B.M., Obrișcă B., Mocanu V., Lupușoru M., Ismail G. (2024). Nephrotic Syndrome: From Pathophysiology to Novel Therapeutic Approaches. Biomedicines.

[6] Wendt R., Sobhani A., Diefenhardt P., Trappe M., Völker L.A. (2024). An Updated Comprehensive Review on Diseases Associated with Nephrotic Syndromes. Biomedicines.

[7] Espi M., Koppe L., Fouque D., Thaunat O. (2020). Chronic Kidney Disease-Associated Immune Dysfunctions: Impact of Protein-Bound Uremic Retention Solutes on Immune Cells. Toxins.

[8] Cohen G. (2020). Immune Dysfunction in Uremia 2020. Toxins.

[9] Steiger S., Rossaint J., Zarbock A., Anders H.J. (2022). Secondary Immunodeficiency Related to Kidney Disease (SIDKD)-Definition, Unmet Need, and Mechanisms. J. Am. Soc. Nephrol..

[10] Gupta S., Pepper R.J., Ashman N., Walsh S.B. (2019). Nephrotic Syndrome: Oedema Formation and Its Treatment With Diuretics. Front. Physiol..

[11] Cąkała-Jakimowicz M., Domaszewska-Szostek A., Puzianowska-Kuznicka M. (2023). Interruption of Lymph Flow Worsens the Skin Inflammation Caused by Saprophytic *Staphylococcus epidermidis*. Biomedicines.

[12] Verma P.R., Patil P. (2024). Nephrotic Syndrome: A Review. Cureus.

[13] Seitz-Polski B., Audard V., Ghiggeri G.M., Tomas N.M. (2022). Editorial: Immune dysfunction in nephrotic syndrome—Recent advances and new roads ahead. Front. Immunol..

[14] Yuan Y., Arcucci V., Levy S.M., Achen M.G. (2019). Modulation of Immunity by Lymphatic Dysfunction in Lymphedema. Front. Immunol..

[15] Arriaga Escamilla D., Lakhani A., Antony S., Salazar Villegas K.N., Gupta M., Ramnath P., Murillo Pineda M.I., Bedor A., Banegas D., Calderon Martinez E. (2024). Dermatological Manifestations in Patients With Chronic Kidney Disease: A Review. Cureus.

[16] Touaitia R., Mairi A., Ibrahim N.A., Basher N.S., Idres T., Touati A. (2025). *Staphylococcus aureus*: A Review of the Pathogenesis and Virulence Mechanisms. Antibiotics.

[17] Lacey K.A., Mulcahy M.E., Towell A.M., Geoghegan J.A., McLoughlin R.M. (2019). Clumping factor B is an important virulence factor during *Staphylococcus aureus* skin infection and a promising vaccine target. PLoS Pathog..

[18] Di Bella S., Marini B., Stroffolini G., Geremia N., Giacobbe D.R., Campanile F., Bartoletti M., Alloisio G., Di Risio L., Viglietti G. (2025). The virulence toolkit of *Staphylococcus aureus*: A comprehensive review of toxin diversity, molecular mechanisms, and clinical implications. Eur. J. Clin. Microbiol. Infect. Dis..

[19] Olaniyi R.O., Pancotto L., Grimaldi L., Bagnoli F. (2018). Deciphering the Pathological Role of Staphylococcal α-Toxin and Panton-Valentine Leukocidin Using a Novel Ex Vivo Human Skin Model. Front. Immunol..

[20] Saeed K., Gould I., Esposito S., Ahmad-Saeed N., Ahmed S.S., Alp E., Bal A.M., Bassetti M., Bonnet E., Chan M. (2018). Panton-Valentine leukocidin-positive *Staphylococcus aureus*: A position statement from the International Society of Chemotherapy. Int. J. Antimicrob. Agents.

[21] Rentinck M.N., Krüger R., Hoppe P.A., Humme D., Niebank M., Pokrywka A., Stegemann M., Kola A., Hanitsch L.G., Leistner R. (2021). Skin infections due to Panton-Valentine leukocidin (PVL)-producing *S. aureus*-Cost effectiveness of outpatient treatment. PLoS ONE.

[22] Aoki A., Hatamiya Y., Fukamatsu H., Sugiyama S., Yamamoto T., Aoyama Y. (2025). Panton-Valentine Leukocidin-Producing Community-Acquired Methicillin-Resistant *Staphylococcus aureus* in Skin and Soft Tissue Infections: Clinical and Epidemiological Insights From Japan. J. Dermatol..

[23] Geeta Sai G., Devi S., Jameela Wahab A. (2024). Unusual Presentation of Staphylococcal Scalded Skin Syndrome in an Elderly Patient With Acute Kidney Injury: A Case Report. Cureus.

[24] Kwiecinski J.M., Kratofil R.M., Parlet C.P., Surewaard B.G.J., Kubes P., Horswill A.R. (2021). *Staphylococcus aureus* uses the ArlRS and MgrA cascade to regulate immune evasion during skin infection. Cell Rep..

[25] Crosby H.A., Schlievert P.M., Merriman J.A., King J.M., Salgado-Pabón W., Horswill A.R. (2016). The *Staphylococcus aureus* Global Regulator MgrA Modulates Clumping and Virulence by Controlling Surface Protein Expression. PLoS Pathog..

[26] Crosby H.A., Keim K., Kwiecinski J.M., Langouët-Astrié C.J., Oshima K., LaRivière W.B., Schmidt E.P., Horswill A.R. (2024). Host-derived protease promotes aggregation of *Staphylococcus aureus* by cleaving the surface protein SasG. mBio.

[27] Westgeest A.C., Schippers E.F., Delfos N.M., Visser L.G., de Fijter J.W., de Boer M.G.J., Lambregts M.M.C. (2022). Acute kidney injury in *Staphylococcus aureus* bacteremia. Eur. J. Clin. Microbiol. Infect. Dis..

[28] Grothe C., Taminato M., Belasco A., Sesso R., Barbosa D. (2016). Prophylactic treatment of chronic renal disease in patients undergoing peritoneal dialysis and colonized by *Staphylococcus aureus*: A systematic review and meta-analysis. BMC Nephrol..

[29] Eryilmaz Eren E., Karagöz N., Saatçi E., Çelik İ., Alp Meşe E. (2025). A Six-Year Surveillance of Nasal Methicillin-Resistant *Staphylococcus aureus* Colonization on Intensive Care Unit Admission: Do We Need Screening?. Infect. Dis. Rep..

[30] Moin S., Salman B., Ahmad A. (2024). *Staphylococcus aureus* Bacteraemia in Patients with Chronic Kidney Disease: Single-Centre Data from Pakistan. Eur. Med. J..

[31] Brown N.M., Goodman A.L., Horner C., Jenkins A., Brown E.M. (2021). Treatment of methicillin-resistant *Staphylococcus aureus* (MRSA): Updated guidelines from the UK. JAC Antimicrob. Resist..

[32] Liu C., Bayer A., Cosgrove S.E., Daum R.S., Fridkin S.K., Gorwitz R.J., Kaplan S.L., Karchmer A.W., Levine D.P., Murray B.E. (2011). Clinical practice guidelines by the infectious diseases society of america for the treatment of methicillin-resistant *Staphylococcus aureus* infections in adults and children. Clin. Infect. Dis..

[33] Stout L., Stephens M., Hashmi F. (2023). Purulent Skin and Soft Tissue Infections, Challenging the Practice of Incision and Drainage: A Scoping Review. Nurs. Res. Pract..

[34] Sartelli M., Coccolini F., Kluger Y., Agastra E., Abu-Zidan F.M., Abbas A.E.S., Ansaloni L., Adesunkanmi A.K., Augustin G., Bala M. (2022). WSES/GAIS/WSIS/SIS-E/AAST global clinical pathways for patients with skin and soft tissue infections. World J. Emerg. Surg..

[35] Maraki S., Mavromanolaki V.E., Stafylaki D., Iliaki-Giannakoudaki E., Hamilos G. (2023). In Vitro Activities of Ceftobiprole, Dalbavancin, Tedizolid and Comparators against Clinical Isolates of Methicillin-Resistant *Staphylococcus aureus* Associated with Skin and Soft Tissue Infections. Antibiotics.

[36] Liu Q., He D., Wang L., Wu Y., Liu X., Yang Y., Chen Z., Dong Z., Luo Y., Song Y. (2024). Efficacy and Safety of Antibiotics in the Treatment of Methicillin-Resistant *Staphylococcus aureus* (MRSA) Infections: A Systematic Review and Network Meta-Analysis. Antibiotics.

[37] Patel D., Brown M.L., Edwards S., Oster R.A., Stripling J. (2023). Outcomes of Daptomycin Plus Ceftaroline Versus Alternative Therapy for Persistent Methicillin-resistant *Staphylococcus aureus* (MRSA) Bacteraemia. Int. J. Antimicrob. Agents.

[38] Gatti M., Viaggi B., Rossolini G.M., Pea F., Viale P. (2023). Targeted Therapy of Severe Infections Caused by *Staphylococcus aureus* in Critically Ill Adult Patients: A Multidisciplinary Proposal of Therapeutic Algorithms Based on Real-World Evidence. Microorganisms.

[39] Górczyńska-Kosiorz S., Lejawa M., Goławski M., Tomaszewska A., Fronczek M., Maksym B., Banach M., Osadnik T. (2024). The Impact of Haplotypes of the *FTO* Gene, Lifestyle, and Dietary Patterns on BMI and Metabolic Syndrome in Polish Young Adult Men. Nutrients.

[40] Yoshizaki S., Shimizu K. (2025). Fulminant Methicillin-Sensitive *Staphylococcus aureus* Pneumonia in a Steroid-Treated Patient With End-Stage Renal Disease: A Rapidly Fatal Case. Cureus.

[41] Davis J.S., Petersiel N., Tong S.Y.C. (2022). How I manage a patient with MRSA bacteraemia. Clin. Microbiol. Infect..

[42] Ghasemiyeh P., Vazin A., Zand F., Haem E., Karimzadeh I., Azadi A., Masjedi M., Sabetian G., Nikandish R., Mohammadi-Samani S. (2022). Pharmacokinetic assessment of vancomycin in critically ill patients and nephrotoxicity prediction using individualized pharmacokinetic parameters. Front. Pharmacol..

[43] Ruiz-Ramos J., Gras-Martín L., Ramírez P. (2023). Antimicrobial Pharmacokinetics and Pharmacodynamics in Critical Care: Adjusting the Dose in Extracorporeal Circulation and to Prevent the Genesis of Multiresistant Bacteria. Antibiotics.

[44] Rybak M.J., Le J., Lodise T.P., Levine D.P., Bradley J.S., Liu C., Mueller B.A., Pai M.P., Wong-Beringer A., Rotschafer J.C. (2020). Therapeutic monitoring of vancomycin for serious methicillin-resistant *Staphylococcus aureus* infections: A revised consensus guideline and review by the American Society of Health-System Pharmacists, the Infectious Diseases Society of America, the Pediatric Infectious Diseases Society, and the Society of Infectious Diseases Pharmacists. Am. J. Health Syst. Pharm..

[45] Xiao Q., Zhang H., Wu X., Qu J., Qin L., Wang C. (2022). Augmented Renal Clearance in Severe Infections-An Important Consideration in Vancomycin Dosing: A Narrative Review. Front. Pharmacol..

[46] Ishigo T., Matsumoto K., Yoshida H., Tanaka H., Ibe Y., Fujii S., Fukudo M., Fujihara H., Yamaguchi F., Ebihara F. (2024). Relationship between nephrotoxicity and area under the concentration-time curve of vancomycin in critically ill patients: A multicenter retrospective study. Microbiol. Spectr..

[47] Song X., Han M. (2022). Pharmacokinetic/Pharmacodynamic Target Attainment of Vancomycin, at Three Reported Infusion Modes, for Methicillin-Resistant *Staphylococcus aureus* (MRSA) Bloodstream Infections in Critically Ill Patients: Focus on Novel Infusion Mode. Front. Cell. Infect. Microbiol..

[48] Guarino M., Perna B., Cesaro A.E., Maritati M., Spampinato M.D., Contini C., De Giorgio R. (2023). 2023 Update on Sepsis and Septic Shock in Adult Patients: Management in the Emergency Department. J. Clin. Med..

[49] Kimmig A., Hagel S., Weis S., Bahrs C., Löffler B., Pletz M.W. (2021). Management of *Staphylococcus aureus* Bloodstream Infections. Front. Med..

[50] Pata R.K., Bastola C., Nway N., Patel M.J., Adhikari S. (2021). Augmented Renal Clearance in a Case of Sepsis Leading to Vancomycin Failure Despite Increasing Dose As per the Estimated Glomerular Filtration Rate. Cureus.

[51] Ishigami J., Grams M.E., Chang A.R., Carrero J.J., Coresh J., Matsushita K. (2017). CKD and Risk for Hospitalization With Infection: The Atherosclerosis Risk in Communities (ARIC) Study. Am. J. Kidney Dis..

[52] Nguyen D.B., Lessa F.C., Belflower R., Mu Y., Wise M., Nadle J., Bamberg W.M., Petit S., Ray S.M., Harrison L.H. (2013). Invasive methicillin-resistant *Staphylococcus aureus* infections among patients on chronic dialysis in the United States, 2005–2011. Clin. Infect. Dis..

[53] Naghavi M., Vollset S.E., Ikuta K.S., Swetschinski L.R., Gray A.P., Wool E.E., Aguilar G.R., Mestrovic T., Smith G., Han C. (2024). Global burden of bacterial antimicrobial resistance 1990–2021: A systematic analysis with forecasts to 2050. Lancet.

[54] Singh K., Raju V., Nikalji R., Jawale S., Patel H., Ahdal J., Jain R. (2019). MRSA Infections in Patients With Renal Disorders: A Review. World J. Nephrol. Urol..

[55] Liu F., Guan Z., Liu Y., Li J., Liu C., Gao Y., Ma Y., Feng J., Shen B., Yang G. (2021). Identification of a Human Anti-Alpha-Toxin Monoclonal Antibody Against *S. aureus* Infection. Front. Microbiol..

[56] Tkaczyk C., Hamilton M.M., Sadowska A., Shi Y., Chang C.S., Chowdhury P., Buonapane R., Xiao X., Warrener P., Mediavilla J. (2016). Targeting Alpha Toxin and ClfA with a Multimechanistic Monoclonal-Antibody-Based Approach for Prophylaxis of Serious *S. aureus* Disease. mBio.

[57] Touati A., Ibrahim N.A., Idres T. (2025). Disarming *Staphylococcus aureus*: Review of Strategies Combating This Resilient Pathogen by Targeting Its Virulence. Pathogens.

